# Efficiency of sFlt-1/PlGF Ratio in Preeclampsia Diagnosis

**DOI:** 10.3390/medicina58091196

**Published:** 2022-09-01

**Authors:** Anca-Florina Tataru-Copos, Mircea Ioachim Popescu, Romina Murvai, Amin El Kharoubi

**Affiliations:** 1 Doctoral School of Biomedical Sciences, Faculty of Medicine and Pharmacy, University of Oradea, 410087 Oradea, Romania; 2 Cardiology Department, Faculty of Medicine and Pharmacy, University of Oradea, 410087 Oradea, Romania

**Keywords:** preeclampsia, sFlt-1/PlGF, proteinuria

## Abstract

*Background and Objectives*: Preeclampsia is a health issue characterized by a new onset of hypertension after 20 weeks of gestation and proteinuria. This is a multiple organ disorder and is associated with significant maternal and fetal mortality. *Material and Methods*: The study is a prospective one and included 69 pregnant women (17 with hypertension without criteria for PE, 26 with severe PE and 26 with moderate PE) with an age of gestation between 24 and 40 weeks. Subjects were chosen from those who referred to the Oradea County Emergency Clinical Hospital, Department of Obstetrics-Gynecology between January 2020 and December 2022. We collected other characteristics from observation sheets and from patients and we measured the sFlt-1/PlGF ratio after 20 weeks of pregnancy if patients presented with suspected preeclampsia. All the results were collected in Excel analysis by SPSS. *Results*: In our study, 37.68% had severe preeclampsia, the same percentage had moderate PE and 24.63% presented only with hypertension. The mean of sFLT-1/PlGF for severe preeclampsia was 78.282 ng/mL, and for moderate, it was 50.154 ng/mL. For those who did not have criteria for preeclampsia, it was 29.076 ng/mL. When we compared the values of sFLT-1/PlGF in moderate PE and hypertension, we found that there was a statistically significant difference between this two, and the same conclusion was also obtained for severe PE and hypertension and for severe and moderate PE. *Conclusions*: This marker can be useful for improving the outcomes for pregnant women with preeclampsia. In addition, for newborns, sFlt-1/PlGF can be helpful because we can correctly and promptly manage a patient affected by this disease before 34 weeks of pregnancy. Our study demonstrates that the correlation between the values of sFlt-1/PlGF and the type of preeclampsia are positive; thus, if the values are high, the pregnant woman likely will develop severe preeclampsia with early onset. In addition, the sFlt-1/PlGF ratio has the highest accuracy for differentiating PE patients from pregnant women who did not develop sign and symptoms for preeclampsia. Our results are in line with the conclusions of other studies that researched the association between sFlt-1/PlGF and clinical diagnosis of preeclampsia.

## 1. Introduction

Preeclampsia is a multisystem disorder characterized by a new onset of hypertension after 20 weeks of gestation and proteinuria or signs of damage to another maternal organ [1]. This is a health issue because preeclampsia is a major cause of maternal morbidity and is associated with unfavorable fetal outcomes such as intra-uterine growth restriction, preterm birth, placental abruption, fetal distress and fetal death in utero [2].

The diagnosis of preeclampsia is based on the criteria of the American College of Obstetrics and Gynecology (ACOG): elevated blood pressure: systolic ≥140 mm Hg or diastolic ≥90 mm Hg, two occasions, 4 h apart in previously normotensive woman; for those who are hypertensive systolic ≥160 mm Hg or diastolic ≥110 mm Hg, AND Proteinuria: ≥300 mg/24 h urine collection, or protein/creatinine ≥0.3, or dipstick reading = 1+, OR severe features: systolic blood pressure ≥160 mm Hg or diastolic ≥110 mm Hg, two occasions, 4 h apart on bedrest; thrombocytopenia (<100,000 μL); liver function tests 2× normal or severe persistent right upper quadrant or epigastric pain; serum creatinine concentration >1.1 mg/dL or doubling of creatinine in the absence of other renal disease; pulmonary edema; new-onset cerebral or visual symptoms [3]. In addition, from ACOG, preeclampsia classified as moderate or severe depending on the levels of blood pressure, proteinuria or other complications. Based on the onset, preeclampsia can be classified as early onset (before 34 weeks of gestation) and late onset (after 34 weeks of gestation).

The pathogenesis of preeclampsia is complex but placental implantation with abnormal trophoblastic invasion of uterine vessels is a major cause followed by an excess of antiangiogenic factors [4]. Antiangiogenic factors include soluble fms-like tyrosine kinase I receptor (sFlt-1) (otherwise known as soluble VEGF receptor type I) and soluble endoglin (sEng) and proangiogenic factors are vascular endothelial growth factor (VEGF) and placental growth factor (PlGF). It was demonstrated that in patients with this disease, the level of sFlt-1 increased and PlGF decreased [5,6]. The ratio sFlt-1/PlGF can be a predictor of preeclampsia before the onset of this (within 4 weeks) [7]. The alterations of these two are more relevant in early-onset rather than late-onset disease and are associated with the severity of the clinical disorder. In some cases, the modifications can occur before the symptoms of preeclampsia [8].

The classification of patients with suspected preeclampsia is difficult and effective care needs the identification and referral of women at high risk. It was demonstrated that the value of the sFlt-1/PlGF ratio cutoff of 38 is useful for the short-term prediction of preeclampsia, maternal/fetal adverse outcomes and preterm delivery [9].

The aim of the study was to evaluate the cut-off values for sFlt-1/Plgf in preeclampsia and the difference between early versus late-onset of the disease and severe versus moderate preeclampsia. This study did not include a control group comprising patients without preeclampsia because we wanted to demonstrate the differences between different groups with this disease.

## 2. Material and Methods

The study is a prospective one and included 69 pregnant women (17 with hypertension without criteria for PE, 26 with severe PE and 26 with moderate PE) with an age of gestation between 24 and 40 weeks. Subjects were chosen from those who were referred to the Oradea County Emergency Clinical Hospital, Department of Obstetrics-Gynecology, between January 2020 and December 2022. From all patients, we obtained informed written consent according to the protocols. We collected other characteristics from observation sheets and from patients, and we measured the sFlt-1/PlGF ratio after 20 weeks of pregnancy if the patients presented suspected preeclampsia. Exclusion criteria: multiple pregnancies, renal disease, collagen vascular diseases, and chronic hypertension. Inclusion criteria: singleton pregnancy, age of gestation (24–40 weeks), hypertension complicated or not with preeclampsia and utero-placental dysfunction (defined by abnormal uterine perfusion with mean pulsatility index >95th percentile in the second trimester and/or bilateral uterine artery notching).

Whole-blood samples were obtained from participants and centrifuged at 3000× *g* for 20 min and separated serum samples stored at −80 °C for ELISA. Levels of sFlt-1 and PlGF were measured using (ELISAR&D System, Human VEGF R1/Flt-1Immunoassay, Catalog Number DVR100B and Human PlGF Immunoassay, Catalog NumberDPG00) according to the procedure provided by the manufacturer. We collected other information from observation sheets and from patients. All results were collected in Excel and analyzed by SPSS. A *p* value of <0.05 defined a statistically significant difference.

The protocol was approved by local ethic committee and institutional review board. All participants provided written informed consent, and the study was conducted in accordance with the principles of the Declaration of Helsinki.

## 3. Results

**Figure 1:**
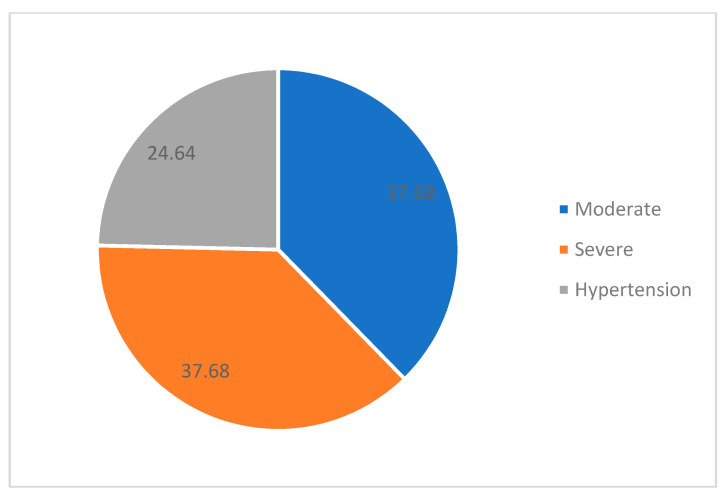
Distribution of preeclampsia.

In our study, 37.68% had severe preeclampsia, the same percentage had moderate PE and only 24.63% had hypertension (Figure 1). The mean of sFLT-1/PlGF for severe preeclampsia was 78.282 ng/mL, and for moderate, it was 50.154 ng/mL. For those who did not fulfill the criteria for preeclampsia, it was 29.076 ng/mL. When comparing the values of sFLT-1/PlGF in moderate PE and hypertension, we found that there is a statistically significant difference between these two (sig = 0.039–Table 1), and the same conclusion was obtained for severe PE and hypertension (sig = 0.000–Table 2) and for severe and moderate PE (sig = 0.020–Table 3). We observed a strong correlation between values of sFlt-1/PlGF and proteinuria (Table 4).

The correlation between the weight of the baby at delivery and the values of sFlt-1/PlGF in each group (moderate PE, severe PE and non-PE) is negative; thus, if the values are higher, the weight of the newborn is smaller (Table 5).

In severe PE, 80.8% of all cases had early onset and 19.2% had late onset. In moderate, only 11.5% of cases had an early onset of the disease and 88.5% had late onset.

The highest incidence of preeclampsia is in the group of 30–34 years old, and the lowest is found in patients under 18 years old (Table 6). The mean age was 29.17, and extreme values were 15 and 44 years old.

## 4. Discussions

We know that antiangiogenic factors such as sFlt-1 increase in pregnant women that are affected by preeclampsia or those that will develop preeclampsia. The level of sFlt-1 increased 4 weeks before clinical manifestations and remained high until the onset of the disease [10,11]. In the same period, the level of PlGF decreased until clinical manifestations [10,11].

Similarly to another study, PROGNOSIS Asia from 2021 showed that the value’s cutoff is 38, and the mean for patients without preeclampsia is 29.076 ng/mL, which was also observed in our study [9].

The values of this ratio are different depending on the type of preeclampsia; it was higher in severe preeclampsia (35–85 for early onset and 35–110 in late onset) [8]; for severe preeclampsia, it was 78.282 ng/mL and 50.154 ng/mL for moderate preeclampsia.

Our study demonstrates that the correlation between the values of sFlt-1/PlGF and the type of preeclampsia is positive; thus, if the values are high, pregnant women will have severe preeclampsia with early onset. In addition, the sFlt-1/PlGF ratio has the highest accuracy for differentiating PE patients from pregnant women who did not develop signs and symptoms of preeclampsia. Our results are in line with the conclusions of other studies that researched the association between sFlt-1/PlGF and the clinical diagnosis of preeclampsia [12,13]. In this manner, this ratio can be very useful for minimize costs and preventing complications for the patient at high risk of developing severe preeclampsia.

In the meantime, the highest sFlt-1/PlGF can cause premature deliveries with low-weight newborns; this marker is important for managing these newborns, who probably will need admission in intensive care units. Complications such as fetal death, fetal growth restriction, results of prematurity and placental abruption given by preeclampsia can be reduced by using this ratio. It is demonstrated that preeclampsia is one of the leading causes of maternal mortality worldwide, and in developed countries, it increases perinatal mortality by five-fold [7,14].

The reduced number of patients included limited this study; we require a larger number to draw a firm conclusion.

## 5. Conclusions

Because the predictive values of sFlt-1/PlGF ratio in preeclampsia are positive, this can be efficient for improving outcomes both for mothers and for newborns. In addition, the costs involved in caring for these patients can be reduced by using these markers because we can predict preeclampsia and the management of these patients can be correct.

## Figures and Tables

**Table 1:** Independent *t*-test for moderate PE and hypertension.

	Levene’s Test for Equality of Variances		*t*-Test for Equality of Means						
	F	Sig	t	df	Sig (2-Tailed)	Mean Difference	Std. Error Difference	95% Confidence Interval of the Difference	
								Lower	Upper
Sflt/plgf Equal variances assumed	4.569	0.039	8.489	41	0.000	21.0774	2.4829	16.0630	26.0918
Sflt/plgf Equal variances not assumed			9.427	40.642	0.000	21.0774	2.2357	16.5610	25.5938

**Table 2:** Independent *t*-test for severe PE and hypertension.

	Levene’s Test for Equality of Variances		*t*-Test for Equality of Means						
	F	Sig	t	df	Sig (2-Tailed)	Mean Difference	Std. Error Difference	95% Confidence Interval of the Difference	
								Lower	Upper
Sflt/plgf Equal variances assumed	23.643	0.000	15.817	41	0.000	49.2158	3.1115	42.9321	55.4996
Sflt/plgf Equal variances not assumed			9.427	40.642	0.000	49.2158	2.6979	43.7509	5454.6808

**Table 3:** Independent *t*-test for severe PE and moderate PE.

	Levene’s Test for Equality of Variances		*t*-Test for Equality of Means						
	F	Sig	t	df	Sig (2-Tailed)	Mean Difference	Std. Error Difference	95% Confidence Interval of the Difference	
								Lower	Upper
Sflt/plgf Equal variances assumed	5.751	0.020	9.468	50	0.000	28.1385	2.9721	22.1689	34.1080
Sflt/plgf Equal variances not assumed			9.468	4.877	0.000	28.1385	2.9721	22.1590	34.1179

**Table 4:** Correlation between proteinuria and values of sFlt-1/PlGF.

Type of Preeclampsia			Sflt-1/plgf	Proteinuria
Moderate	Sflt-1/plgf	Pearson Correlation	1	0.423 *
		Sig (2-tailed)		0.031
		N	26	26
	Proteinuria	Pearson Correlation	0.423 *	1
		Sig (2-tailed)	0.031	
		N	26	26
Hypertension	Sflt-1/plgf	Pearson Correlation	1	0.617 **
		Sig (2-tailed)		0.008
		N	17	17
	Proteinuria	Pearson Correlation	0.617 **	1
		Sig (2-tailed)	0.008	
		N	17	17
Severe	Sflt-1/plgf	Pearson Correlation	1	0.701 **
		Sig (2-tailed)		0.000
		N	26	26
	Proteinuria	Pearson Correlation	0.701 **	1
		Sig (2-tailed)	0.000	
		N	26	26

*. Correlation is significant at the 0.05 level (2-tailed). **. Correlation is significant at the 0.01 level (2-tailed).

**Table 5:** Correlation between newborn weight and values of sFlt-1/PlGF.

Type of Preeclampsia			Sflt-1/plgf	Newborn Weight
Moderate	Sflt-1/plgf	Pearson Correlation	1	−0.119
		Sig (2-tailed)		0.563
		N	26	26
	Newborn weight	Pearson Correlation	−0.119	1
		Sig (2-tailed)	0.563	
		N	26	26
Hypertension	Sflt-1/plgf	Pearson Correlation	1	−0.537 *
		Sig (2-tailed)		0.026
		N	17	17
	Newborn weight	Pearson Correlation	−0.537 *	1
		Sig (2-tailed)	0.026	
		N	17	17
Severe	Sflt-1/plgf	Pearson Correlation	1	−0.507 **
		Sig (2-tailed)		0.010
		N	26	26
	Newborn weight	Pearson Correlation	−0.507 **	1
		Sig (2-tailed)	0.010	
		N	26	26

*. Correlation is significant at the 0.05 level (2-tailed). **. Correlation is significant at the 0.01 level (2-tailed).

**Table 6:** Age distribution.

Age Range (Years)	Number of Cases	Prevalence
<18	6	8.70%
18–24	13	18.84%
25–29	15	21.74%
30–34	16	23.19%
35–39	11	15.94%
>40	8	11.59%

## Data Availability

Not applicable.
